# Dexamethasone regime and clinical outcomes in children hospitalized with croup: A cohort study

**DOI:** 10.1002/jhm.13542

**Published:** 2024-11-01

**Authors:** David D'Arienzo, Muhammadhasan Nasser, Peter J. Gill, Cornelia M. Borkhoff, Patricia C. Parkin, Sanjay Mahant

**Affiliations:** ^1^ Department of Paediatrics, Montreal Children's Hospital McGill University Montreal Quebec Canada; ^2^ Division of Paediatric Medicine Hospital for Sick Children Toronto Ontario Canada; ^3^ Department of Paediatrics, Temerty Faculty of Medicine University of Toronto Toronto Ontario Canada; ^4^ Temerty Faculty of Medicine University of Toronto Toronto Ontario Canada; ^5^ Pediatric Outcomes Research Team Hospital for Sick Children Toronto Ontario Canada; ^6^ Child Health Evaluative Sciences Hospital for Sick Children Research Institute Toronto Ontario Canada; ^7^ Institute of Health Policy, Management and Evaluation, Dalla Lana School of Public Health University of Toronto Toronto Ontario Canada

## Abstract

**Background:**

High‐quality trial evidence supports the use of one dose of dexamethasone in the outpatient management of croup; however, there are no inpatient trials, and the optimal treatment regimen for the inpatient management of croup remains uncertain. Significant practice variability exists in the corticosteroid treatment of children hospitalized for croup.

**Objective:**

To evaluate the association of dexamethasone treatment regimen (1 vs. >1 dose) with hospital length of stay (LOS) and 30‐day return to emergency department (ED) visits among children hospitalized for croup.

**Methods:**

A cohort study of children hospitalized for croup at a children's hospital between 2010 and 2022. Children less than 10 years old, without known airway anomalies and who received dexamethasone for croup treatment were included. Children who received 1 dose versus >1 dose of dexamethasone were compared. Propensity score analyses, using inverse probability of treatment weighting, were conducted to estimate the treatment effects of dexamethasone regimen on hospital LOS and all‐cause 30‐day return to ED visit.

**Results:**

Of 471 children hospitalized for croup, 229 (49%) received 1 dose of dexamethasone; 242 (51%) received >1 dose. In the propensity‐weighted analyses, children receiving >1 dose of dexamethasone had a longer mean LOS by 59.6 h (95% CI 44.8–74.5, *p* < .001) compared with those receiving >1 dose. There was no statistically significant difference in the odds of all‐cause 30‐day return to ED visit; OR 1.30, (95% CI 0.76–2.22, *p* = .33).

**Conclusions:**

Among children hospitalized for croup, children who received >1 dose of dexamethasone had a longer LOS compared with children who received 1 dose of dexamethasone; however, there was no statistically significant difference in the 30‐day return to ED visits. Randomized clinical trials are needed to determine the optimal dexamethasone treatment regimen for children hospitalized with croup.

## Visual Abstract

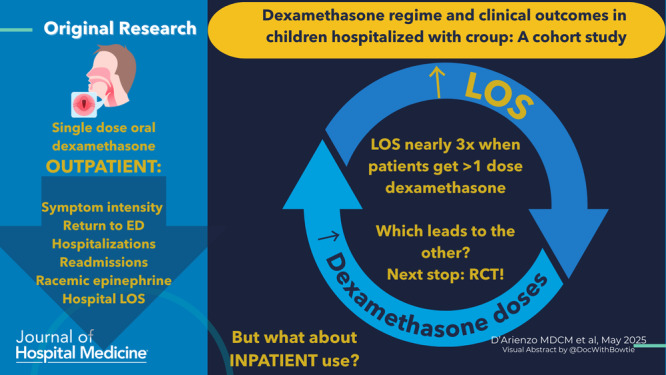

## INTRODUCTION

Croup is a common pediatric respiratory illness with a large burden on the healthcare system. Each year, about 3% of US children are diagnosed with croup, and although it is a self‐limiting condition, it accounts for 5% of emergency department (ED) visits in children under 2 years of age and 7% of all hospitalizations of children younger than 5 years.^1^, ^2^, ^3^, ^4^, ^5^ Croup can be a significant source of morbidity, with 9% of hospitalized children requiring intensive care unit (ICU) admission, and 1%–3% requiring intubation.^6^

A strong evidence base of randomized clinical trials (RCTs) and guidelines support the use of oral dexamethasone for croup management in the outpatient and ED setting. A single dose of oral dexamethasone for outpatient management of croup reduces the intensity of symptoms and decreases the rate of return to ED visits, hospitalizations, and readmissions.^7^, ^8^, ^9^, ^10^ Glucocorticoid treatment has also reduced the need for inhaled racemic epinephrine and decreased length of stay (LOS) in hospital.^10^, ^11^ Despite the strong evidence for glucocorticoids in the outpatient setting, there is a lack of evidence guiding the inpatient management of croup.

Despite the lack of RCTs supporting the clinical effectiveness of multiple doses of dexamethasone on duration of symptoms and healthcare use, children hospitalized for croup are often treated with multiple doses of dexamethasone.^12^ Up to 58% of hospitalized patients receive multiple doses of dexamethasone in some hospitals.^6^ Physicians use multiple doses of dexamethasone in an effort to improve symptoms, decrease LOS in hospital, and reduce return visits to the hospital after discharge.^6^, ^13^, ^14^ In a single‐center retrospective cohort study of children admitted to the ICU, treatment with multiple doses of dexamethasone was not associated with a decrease in the return of croup symptoms.^13^ Perceived benefits of multiple doses of dexamethasone in croup management must also be weighed against the well‐established risks of dexamethasone, including hyperactivity, sleep disturbance, secondary bacterial infection, and vomiting.^15^ The lack of clinical guidelines regarding inpatient management of croup may contribute to variability of care, as well as excess healthcare utilization and cost.

Our objective was to examine the association of dexamethasone treatment regimen, one dose versus multiple doses, with hospital LOS and return to ED visits within 30‐days from hospital discharge.

## METHODS

### Study design and setting

This was a cross‐sectional cohort study of children hospitalized for croup at a tertiary care pediatric hospital between January 1, 2010 and December 31, 2022, in Canada. The data source was the hospital's electronic medical records. Ethics approval was obtained from the institution's Research Ethics Board.

### Study population

Children aged 0–120 months, with the most responsible discharge diagnosis of acute croup (based on *the International Classification of Disease (ICD) 9th and 10th Revision* diagnostic codes) were included (Appendix A). Patients administered oral or intravenous dexamethasone at the index hospitalization (including in the ED) for the treatment of croup were included. Patients were excluded if they (i) were administered a steroid other than dexamethasone, as the mainstay of croup treatment now is dexamethasone; or (ii) if they had a history of an underlying airway anomaly (Appendix B), as it may lead to diagnostic inaccuracy and/or a complicated hospital course.^1^, ^16^ Data were collected from electronic medical records, extracted by two study team members not involved in data analysis.

### Exposure

The exposure variable was dexamethasone dosing regimen: treatment with 1 dose versus >1 dose of dexamethasone during the hospitalization. The sum of dexamethasone doses administered in the hospital, including in the ED, was used to determine the treatment group.

### Outcome

The primary outcome was hospital LOS, measured in hours from time of hospital admission to discharge. The secondary outcome was all‐cause 30‐day return visit to the ED. Hospital LOS is recognized as a priority outcome for caregivers of children with croup.^17^ LOS is also recognized as an important performance metric for both the effectiveness and efficiency of the health care system.^18^, ^19^ As dexamethasone is also known to reduce croup duration of illness, it may also reduce LOS.^15^ The 30‐day return to ED visit is an important quality of care metric and can represent a measure of harm or treatment failure.^20^, ^21^

### Covariates

Potential confounders were identified a priori. Potential confounders included age (months), sex (male/female), presence of chronic disease, history of prematurity (<37 weeks gestation), history of croup, history of intubation, systemic steroid use in the prior 7 days, as well as various investigations and treatments initiated in the ED, including chest X‐ray, neck X‐ray, number of doses of inhaled racemic epinephrine, direct ICU admission, and initiation of intravenous fluids (IVF), supplemental oxygen (O_2_), salbutamol, or antibiotics. The presence of chronic disease was assessed by a physician and defined as a medical condition that would reasonably be expected to last at least 12 months and require specialty pediatric care.^22^ Lower age and prematurity are associated with more severe respiratory outcomes in children with viral respiratory illnesses, likely due to smaller airways.^23^, ^24^ Males with respiratory illness also have poorer health outcomes compared with females.^25^ Patients with chronic disease are at higher risk for greater morbidity with respiratory illnesses, which can impact LOS.^23^, ^26^ Previous croup may indicate an undiagnosed upper airway anomaly and previous intubation may result in acquired upper airway anomalies (i.e., subglottic stenosis), resulting in more severe croup.^27^, ^28^

### Statistical analyses

#### Descriptive statistics

Baseline characteristics, including demographic and clinical characteristics, were compared by treatment group (1 vs. >1 dose of dexamethasone). Data on diagnostic testing, supportive care, and clinical outcomes were collected for the entire cohort. Continuous variables were described using mean and standard deviation for normally distributed variables, median and interquartile range for nonnormally distributed variables. Two‐sided *t* tests or Wilcoxon Rank‐Sum Test were used to compare groups. Categorical variables were described using frequencies and proportions (%) and compared using *χ* ^2^ tests.

#### Propensity score analysis

Propensity scores, with inverse probability treatment weighted (IPTW) analyses, were used to control for potential confounding and ensure the two groups were balanced.^29^ Propensity scores were calculated using multivariable logistic regression, with patients' age, sex, year of hospitalization, chronic disease, history of prematurity, prior croup, prior intubation, systemic steroid use in the prior 7 days, and initiation of investigations/treatments in the ED including chest X‐ray, neck X‐ray, IVF, supplemental O_2_, salbutamol, antibiotics, number of doses of inhaled racemic epinephrine, and direct ICU admission as the independent variables. Weighted samples, using IPTW, were used to estimate the average treatment effect for those administered >1 dose of dexamethasone. Independent samples *t* tests/*χ* ^2^ tests and standardized mean differences were used to assess the balance in baseline characteristics between the two weighted treatment groups.

Two multivariable models were constructed. A linear regression model to assess the association between dexamethasone treatment regimen and LOS. Unadjusted and weighted parameter estimates were calculated, along with the 95% confidence intervals (CIs). We used a logistic regression model to examine the association between dexamethasone treatment regimen and 30‐day return to ED visit. The odds ratio (OR) and 95% CIs were calculated.

Sensitivity analyses were performed to confirm the robustness of the results. Weighted analyses were reported. First, we excluded encounters where it was documented in the chart that the patient received steroids in the 7 days before index hospitalization, and we excluded encounters when ICU was consulted. When evaluating the secondary outcome, we also excluded encounters when dexamethasone was prescribed at discharge. Additionally, to improve the robustness of the results to outliers, a median regression was performed, adjusting for all variables included in the propensity score calculation.

All statistical analysis were performed using SAS software version 9.4 (SAS Institute) and a two‐tailed *p* value < .05 was considered statistically significant.

## RESULTS

### Cohort description

**Figure 1:**
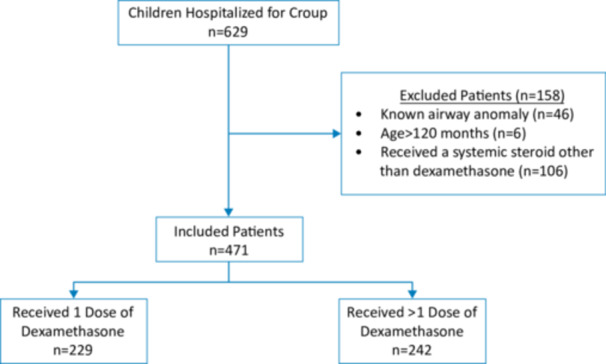
Flowchart of patients hospitalized for croup and included in the study.

A total of 471 children 0–120 months of age were included in the study; 229 (49%) children received 1 dose of dexamethasone, 242 (51%) children received >1 dose of dexamethasone (Figure 1). The study sample had a median (IQR) age of 16^10^, ^11^, ^12^, ^13^, ^14^, ^15^, ^16^, ^17^, ^18^, ^19^, ^20^, ^21^, ^22^, ^23^, ^24^, ^25^ months, 405 (86%) were born at term (≥37 weeks gestation), and 108 (23%) had an underlying chronic condition. The median (IQR) number of doses of dexamethasone received per patient was 1.^1^, ^2^ There were no missing values for our primary outcome (LOS) and exposure, and <1% of observations (*n* = 3) with missing values for our secondary outcome (30‐day return to ED visits). All covariates had <1% missing data except for the covariate on history of intubation, which had 10% missing data (Table 1). Given the rarity and clinical significance of intubation, any missing data for this covariate was considered no history of intubation. Additionally, given the study cohort included only 8 patients (1.7%) that were positive for COVID‐19, adjusting for COVID‐19 infections was not necessary.

**Table 1:** Baseline patient characteristics used in propensity score model.

	Unweighted study population				Weighted study population				
Characteristics	Dex dose = 1 *n* = 229 (49%)	Dex dose >1 *n* = 242 (51%)	SMD^a^	*p* Value	Dex dose = 1 *n* = 205 (49%)	Dex dose >1 *n* = 218 (51%)	SMD^a^	*p* Value	Missing *n* (%)
Age, months median (IQR)	22 (9–28)	20 (10–24)	0.10	.18	15 (8–25)	16 (10–24)	0.008	.93	0
Sex									
Male, *n* (%)	158 (69%)	169 (70%)	0.03	.84	137.2 (67%)	150.3 (69%)	0.04	.52	0
Female, *n* (%)	71 (31%)	73 (30%)			67.8 (33%)	67.7 (31%)			
Presence of chronic disease,^b^ *n* (%)	51 (22%)	57 (24%)	0.04	.74	47.7 (23%)	50.5 (23%)	0.003	.97	0
History of croup, *n* (%)	39 (17%)	56 (23%)	0.14	.10	43.7 (21%)	424.3 (20%)	0.02	.72	1
History of prematurity (<37 weeks), *n* (%)	64 (28%)	89 (35%)	0.07	.49	24.7 (12%)	25.3 (12%)	0.01	.83	1
History of prior intubation, *n* (%)	5 (2%)	19 (8%)	0.26	.005	10.1 (5%)	10.9 (5%)	0.008	.90	47
Systemic steroid use in 7 days prior, *n* (%)	65 (28%)	94 (39%)	0.21	.02	71.0 (35%)	74.9 (34%)	0.006	.93	
Investigations/treatments initiated in the emergency department									
Chest X‐ray	48 (21%)	48 (20%)	0.01	.76	39.2 (19%)	43.3 (20%)	0.02	.79	
Neck X‐ray	44 (19%)	57 (24%)	0.10	.25	42.2 (21%)	46.3 (21%)	0.02	.81	0
Intravenous fluids	55 (24%)	55 (23%)	0.03	.74	48.6 (24%)	50.9 (23%)	0.009	.90	0
Salbutamol	15 (7%)	25 (10%)	0.14	.14	15.8 (8%)	17.7 (8%)	0.02	.80	0
Antibiotics	25 (11%)	27 (11%)	0.005	.93	20.5 (10%)	22.6 (10%)	0.01	.85	0
Supplemental oxygen	10 (4%)	21 (9%)	0.17	.06	13.7 (7%)	14.5 (7%)	0.002	.98	0
Inhaled racemic epinephrine doses, median (IQR)	1 (0–3)	2 (0–3)	0.33	.001	2 (0–3)	2 (0–3)	0.05	.54	0
Direct ICU admission	12	38	0.38	<.001	16.0 (8%)	19.5 (9%)	0.04	.54	0

Abbreviations: Dex, dexamethasone; IQR, interquartile range.

^a^ Standardized mean differences >0.1 denotes meaningful imbalance in the baseline covariate.^30^

^b^ Chronic disease defined as a medical condition that would reasonably be expected to last at least 12 months and require specialty pediatric care.^22^

The diagnostic tests, supportive care provided, and associated outcomes of all study participants are summarized in Table 2. Among the patients hospitalized for croup, 49% had a blood test. The most common blood tests ordered were a complete blood count (46%), electrolytes (43%), and a blood culture (29%). A chest X‐ray (CXR) was performed in 42% of children, neck X‐ray in 35%. The majority of investigations were performed in the ED; complete blood counts (85% done in the ED), electrolytes (52%), blood culture (49%), nasopharyngeal swab (16%), CXR (50%), and neck X‐ray (61%). Antibiotics were initiated for 29% of patients and continued at discharge for 11% of patients. A prescription for dexamethasone was given at discharge in 16% of patients. Intravenous fluids were administered in 48% of patients, while inhaled racemic epinephrine was administered in 83% of patients, and inhaled salbutamol in 19% of patients. The majority of supportive care measures were initiated on the pediatrics wards; antibiotics (39%), IV fluid initiation (49%), salbutamol (48%). Finally, the ICU was consulted in 20% of patients, 16% were admitted to the ICU (89% admitted directly from ED to ICU), 7% were intubated, and there were two patients (0.4%) deaths.

**Table 2:** Investigations, supportive care, and outcomes of children hospitalized for croup.

	Dexamethasone dose = 1 *n* = 229 (49%)	Dexamethasone dose >1 *n* = 242 (51%)	Total *n* = 471
*Investigations*			
Blood tests	95 (42%)	136 (56%)	231 (49%)
Blood culture	58 (25%)	78 (32%)	136 (29%)
Positive blood culture	2 (1%)	4 (2%)	6 (1%)
Nasopharyngeal (NP) swab	66 (29%)	114 (47%)	180 (38%)
Positive NP swab	32 (14%)	57 (24%)	89 (19%)
Chest X‐ray	74 (32%)	125 (52%)	199 (42%)
Neck X‐ray	61 (27%)	105 (44%)	166 (35%)
*Supportive care*			
Antibiotics given in hospital	48 (21%)	88 (36%)	134 (29%)
Antibiotics prescribed at discharge	16 (7%)	35 (14%)	51 (11%)
Dexamethasone prescribed at discharge	39 (17%)	35 (14%)	74 (16%)
Intravenous fluids	95 (42%)	133 (55%)	228 (48%)
Inhaled racemic epinephrine	182 (80%)	211 (87%)	393 (83%)
Inhaled salbutamol	24 (11%)	59 (25%)	83 (18%)
Supplemental O_2_	16 (7%)	59 (25%)	75 (16%)
*Outcomes*			
ICU consultation	14 (7%)	80 (33%)	94 (20%)
ICU admission	10 (4%)	66 (27%)	76 (16%)
Intubation	3 (1%)	32 (13%)	35 (7%)
Mortality	1 (0.4%)	1 (0.4%)	2 (0.4%)

Abbreviations: ICU, intensive care unit; NP, nasopharyngeal.

Baseline characteristics before and after propensity score weighting are presented (Table 1). Standardized mean differences across all covariates were less than 0.1 in the weighted population, indicating good overall balance between groups.^30^ Primary and secondary outcomes in the two groups are summarized in Table 3.

**Table 3:** Primary and secondary outcomes by dexamethasone treatment regimen.

Outcome	Dexamethasone dose = 1 *n* = 229 (49%)	Dexamethasone dose >1 *n* = 242 (51%)	Difference in means (95% CI)	*p* Value
*Unadjusted analyses*				
Length of stay (hours), mean (SD)	35.7 (465.4)	89.6 (98.7)	53.8 (40.3–68.0)	<.001
*Propensity score adjusted analyses*				
Length of stay (hours), mean (SD)	35.3 (42.2)	90.9 (105.5)	59.6 (44.8–74.5)	<.001

	Dexamethasone dose = 1 *n* = 228 (49%)^a^	Dexamethasone dose >1 *n* = 240 (51%)^a^	Odds ratio (95% CI)	***p*** **Value**
*Unadjusted analyses*				
30‐day return to ED visit				
All‐cause, *n* (%)	34 (15%)	29 (12%)	1.30 (0.76–2.22)	.33
Croup‐related, *n* (%)	10 (4%)	14 (6%)	0.76 (0.33–1.76)	.53
*Propensity score adjusted analyses*				
30‐day return to ED visit				
All‐cause, *n* (%)	34 (15%)	29 (12%)	1.39 (0.95–2.02)	.09
Croup‐related, *n* (%)	10 (4%)	14 (6%)	0.79 (0.40–1.78)	.52

Abbreviation: ED, emergency department.

^a^ The children with missing secondary outcome data (30‐day return to ED visit) were excluded from the secondary outcome analysis.

### Association of dexamethasone dosing regimen and hospitalization LOS

Univariate analysis, before weighting on the calculated propensity scores, showed that dexamethasone dosing regimen, 1 versus >1 dose of dexamethasone, was associated with longer LOS. Patients who received >1 dose of dexamethasone had a mean LOS of 53.8 h (95% CI 40.3–68.0, *p* < .001) longer than those who received 1 dose of oral dexamethasone when not adjusting for other variables. After IPTW, using the calculated propensity scores, dexamethasone treatment regimen was associated with longer LOS. LOS was significantly longer among the group that received >1 dose of dexamethasone as compared with those that received 1 dose, by 59.6 h (95% CI 44.8–74.5, *p* < .001) (Table 3). Those that received 1 dose of dexamethasone had a mean (SD) LOS of 35.3 h (42.2); those that received >1 dose of dexamethasone had a mean (SD) LOS of 90.9 h (105.5).

Sensitivity analyses, using propensity score analyses, demonstrated similar results between the association of dexamethasone treatment regimen and LOS. When excluding 159 children that received steroids in the 7 days before index hospitalization, LOS was 58.1 h longer among those that received >1 dose (95% CI 38.9–77.3, *p* < .001). Upon excluding 94 children that had an ICU consultation, dexamethasone treatment regimen was again associated with LOS (39.9 h longer among those that received >1 dose, 95% CI 26.3–53.6, *p* < .001) in the weighted analysis. Additionally, median regression, when adjusting for all potential confounders, demonstrated that the median LOS was 35.7 h (95% CI 22.9–46.0, *p* < .001) longer in the cohort that received >1 dose of dexamethasone compared with those that received 1 dose.

### Association of dexamethasone and 30‐day return to ED visit

Among patients who received 1 dose of dexamethasone, 34 patients (15%) had an all‐cause 30‐day return to ED visit; while for those who received >1 dose of dexamethasone, 29 patients (12%) had an all‐cause 30‐day return to ED visit. In adjusted analysis, before weighting on the calculated propensity scores, there was no increased odds in a 30‐day return to ED visit among children who received >1 dose of dexamethasone compared with those who received 1 dose (OR 1.30, 95% CI 0.76–2.22, *p* = .33). Similarly, after IPTW, there was no increased odds in a 30‐day return to ED visit between dexamethasone treatment regimens (OR 1.39, 95% CI 0.95–2.02 *p* = .09). Indications for return to ED visit, by treatment group, are presented in Appendix C. Twenty‐four children (5%) returned to the ED for croup‐related symptoms—10 patients who received 1 dose of dexamethasone, 14 patients who received >1 dose.

In sensitivity analyses, there was no association between dexamethasone treatment regimen and 30‐day return to ED visit when (i) excluding children who had reportedly received steroids in the 7 days before index hospitalization (OR 1.13, 95% CI 0.70–1.81, *p* = .62), (ii) excluding children where ICU was consulted (OR 1.02, 95% CI 0.67–1.56, *p* = .93), and (iii) excluding patients who were prescribed an additional dose of dexamethasone at discharge (OR 1.06, 95% CI 0.70–1.59, *p* = .91).

## DISCUSSION

In this cohort study from a large Canadian children's hospital, 51% of children hospitalized for croup received >1 dose of dexamethasone despite little evidence supporting the practice. Using propensity‐weighted analyses, we found that hospital LOS was 59.6 h longer in children who received >1 dose of dexamethasone compared with those children who received 1 dose. However, we found no significant difference in 30‐day return visits to the ED in those children who received 1 versus >1 dose of dexamethasone.

It is important to note that the finding of a longer LOS with >1 dose of dexamethasone may be explained by residual confounding. It is possible that sicker patients were given >1 dose of dexamethasone and, due to illness severity, were observed in the hospital longer until clinical improvement. Similarly, those that were observed in hospital for longer periods of time may have been given additional doses of dexamethasone. Clinicians may also choose to extend LOS among those who receive multiple doses of dexamethasone to ensure a response to treatment and to monitor for symptom recurrence. Our study highlights the need for a RCT, which can overcome the confounding bias in observational study designs, to assess the optimal corticosteroid treatment regimen for the inpatient management of croup.

The 30‐day return to ED visit is an important quality of care metric and can highlight care deficiencies (i.e., ineffective treatment) and safety concerns.^21^ In our population, 13% (*n* = 63) children experienced a 30‐day return to ED visit, 15% (*n* = 34) in the >1 dose of dexamethasone group and 12% (*n* = 29) in those who received 1 dose of dexamethasone. Given that there was no statistically significant difference in 30‐day return to ED visits between the two treatment groups, it is possible that >1 dose of dexamethasone may be overtreatment, potentially resulting in undue adverse effects (although unlikely serious), without clinically significant improvement. Conversely, it is possible that the sicker patients with croup responded to the additional dose(s) of dexamethasone and had similar ED‐revisit rates to those children who only required 1 dose.

Multiple doses of dexamethasone was found to be associated with an increased LOS in patients hospitalized with croup and not associated with 30‐day return to ED visits, in a previous study.^12^ Tyler et al. used regression analyses to demonstrate that children who that received >1 dose of dexamethasone had a LOS of 27 h longer than those who received 1 dose and showed no association with 30‐day return to ED visits.^12^ Our study adds to this literature as it accounted for additional clinically relevant baseline characteristics, had a larger sample size, and utilized propensity score weighting, a more favorable approach to traditional regression analyses when estimating causal effects using observational data.^29^, ^31^

Given the lack of strong evidence and guidelines, there is significant practice variation in the inpatient management of croup.^32^ A large US study, utilizing administrative data, demonstrated wide variability in the management and outcomes of patients hospitalized for croup, including treatment and investigations.^6^ This variability was also noted in our single center Canadian study. Furthermore, we found that most imaging and laboratory testing is ordered in the ED. Although numerous quality improvement initiatives are underway to streamline inpatient croup management, ED‐related interventions may have the greatest impact.

## LIMITATIONS

This study has several limitations. First, the study population only included patients from one center. However, data were collected over 12‐years and the sample size was relatively large, compared with other pediatric studies investigating inpatient croup. Second, ED revisits are rare occurrences; we were unable to capture ED revisits to other health centers and we may not have been sufficiently powered to detect small differences in 30‐day return to ED visit rates. Third, we did not examine timing of the second dexamethasone dose. Fourth, we did not measure the outcome of rebound croup, which may be an appropriate outcome when assessing the effect of dexamethasone treatment.^13^ Fifth, there may be physician‐level preference for prescribing additional doses of dexamethasone, which could not be accounted for due to limitations of the electronic health record and available data. Additionally, we acknowledge despite propensity analyses, we may not be fully accounting for residual confounding by indication. As such, we can only report association; an RCT is needed to evaluate causality.

## CONCLUSIONS

Among children hospitalized for croup, the use of >1 dose of dexamethasone compared with 1 dose of dexamethasone was associated with increased LOS, with no difference in the 30‐day return visit to ED. This study highlights the need for an RCT to determine the optimal dexamethasone dosing regimen for children hospitalized for croup.

## CONFLICT OF INTEREST STATEMENT

The authors declare no conflict of interest.

## ETHICS STATEMENT

Ethics approval was obtained from the Hospital for Sick Children Research Ethics Board.

## Data Availability

Deidentified individual participant data will not be made available.

## APPENDIX A

See Table A1.

**Table A1:** *International Classification of Diseases 9th and 10th Revisions* (ICD‐9, ICD‐10) corresponding to a diagnosis of croup.

*ICD‐9 codes*	
Acute laryngitis and tracheitis	464
Acute laryngitis	464.0
Acute tracheitis	464.1
Acute laryngotracheitis	464.2
Croup	464.4
*ICD‐10 codes*	
Acute laryngitis and tracheitis	J04
Acute laryngitis	J04.0
Acute tracheitis	J04.1
Acute laryngotracheitis	J04.2
Acute obstructive laryngitis	J05

Abbreviation: ICD, International Classification of Diseases.

## APPENDIX B

See Table B1.

**Table B1:** *International Classification of Diseases 9th and 10th Revisions* (ICD‐9, ICD‐10) corresponding to a diagnosis of anatomical airway anomalies.

*ICD‐9 codes*	
Other diseases of upper respiratory tract	478
Congenital anomalies of the respiratory system	748
Other specified congenital anomalies of mouth and pharynx	750.2
Other congenital anomalies of the face or neck	744.8
Tracheo‐Oesophageal Fistula, Oesophageal Atresia, and Stenosis	750.3
Tracheosotomy	V44.0
*ICD‐10 codes*	
Other specified disease of upper respiratory tract	J39.8
Congenital malformations of larynx	Q31
Congenital malformations of lung	Q33
Congenital malformations of the trachea and bronchus	Q32
Congenital malformations of the respiratory system	Q34
Postprocedural subglottic stenosis	J95.5
Tracheostomy	Z93.0

Abbreviation: ICD, International Classification of Diseases.

## APPENDIX C

See Table C1.

**Table C1:** Indications for 30‐day return to ED visit following hospitalization for croup by dexamethasone treatment regimen.

Indication for return to ED visit	Dexamethasone dose = 1 (*n* = 33)	Dexamethasone dose >1 (*n* = 30)
Croup	10 (30%)	14 (47%)
Required readmission	6 (18%)	10 (33%)
Cough/congestion/fever	15 (46%)	7 (23%)
Vomiting	4 (12%)	4 (13%)
Other	4 (12%)	5 (17%)
